# Small-Angle Scattering and Multifractal Analysis of DNA Sequences

**DOI:** 10.3390/ijms21134651

**Published:** 2020-06-30

**Authors:** Eugen Mircea Anitas

**Affiliations:** 1 Joint Institute for Nuclear Research, Dubna 141980, Russia; anitas@theor.jinr.ru; 2 Horia Hulubei, National Institute of Physics and Nuclear Engineering, 077125 Bucharest-Magurele, Romania

**Keywords:** DNA sequences, multifractals, small-angle scattering, multiplicative cascades

## Abstract

The arrangement of A, C, G and T nucleotides in large DNA sequences of many prokaryotic and eukaryotic cells exhibit long-range correlations with fractal properties. Chaos game representation (CGR) of such DNA sequences, followed by a multifractal analysis, is a useful way to analyze the corresponding scaling properties. This approach provides a powerful visualization method to characterize their spatial inhomogeneity, and allows discrimination between mono- and multifractal distributions. However, in some cases, two different arbitrary point distributions, may generate indistinguishable multifractal spectra. By using a new model based on multiplicative deterministic cascades, here it is shown that small-angle scattering (SAS) formalism can be used to address such issue, and to extract additional structural information. It is shown that the box-counting dimension given by multifractal spectra can be recovered from the scattering exponent of SAS intensity in the fractal region. This approach is illustrated for point distributions of CGR data corresponding to *Escherichia coli*, *Phospholamban* and *Mouse mitochondrial* DNA, and it is shown that for the latter two cases, SAS allows extraction of the fractal iteration number and the scaling factor corresponding to “ACGT” square, or to recover the number of bases. The results are compared with a model based on multiplicative deterministic cascades, and respectively with one which takes into account the existence of forbidden sequences in DNA. This allows a classification of the DNA sequences in terms of random and deterministic fractals structures emerging in CGR.

## 1. Introduction

Understanding the correlations between DNA structure and its functions is one of the fundamental challenges in modern biology, with important implications in various biological processes, such as in replication or transcription [1]. Basically, eukaryotic DNA has a hierarchical organization in which the primary structure consists of a sequence of four nucleotides, i.e., adenine (A), thymine (T), guanine (G) and cytosine (C), arranged in two complimentary polynucleotide strands in the form of a double helix. In this configuration, each type of nucleotide on one strands, bonds with just one type of nucleotide on the other strand, according to base pairing rules: A with T, and C with G. At a higher level, DNA is tightly packed into nucleosomes (almost two turns of DNA wrapped around histone protein), separated by additional DNA fragments. Furthermore, the nucleosomes are arranged into a chromatin fiber, which forms loops, then chromatin domains, and finally chromosomes [2]. As such, the number of base pairs involved increases from about $10^{2}$ bp (for short regions of DNA double helix), up to about $10^{2}$ Mbp (for chromosomes).

It is argued that the primary structure contributes, through gene positions and transcriptional activity to the organization of chromatin at all scales, although the precise influence remains controversial [1]. Up to some extent, this may be linked to the existence of long-range correlations of nucleotide distributions [3,4,5,6,7]. The nature and origin of such correlations is intensively studied in the literature by performing various types of statistical analysis on DNA sequences [8,9,10,11,12,13]. One of the most accurate method in revealing the existence of power-law correlations with specific scale-invariance properties is the wavelet transform modulus maxima (WTMM) [13]. This method considers analyzing wavelets that make the wavelet transform microscope blind to low-frequency trends, and can reveal the hierarchy that governs the spatial distribution of multifractal measures [1].

More recently, with the advent of bioinformatics, numerical representation of DNA sequences has become an important approach in analyzing long-range correlations in big data sets as generated from high-throughput methods for sequencing. To this aim, a popular method, which is gaining an increased interest is the chaos game representation (CGR), introduced by Jeffrey in Ref. [14]. This is an iterative mapping technique which assigns to each nucleotide unique coordinates in 2D space, and it allows a visual representation of both local and global patterns in DNA sequences, revealing previously unknown structures [14]. One of the most important property, which makes CGR very useful in numerical encoding is that it gives a one-to-one representation, i.e., given a CGR point in the plane, one can trace back to the origin of the sequence, and therefore the original DNA sequence can always be reconstructed [15]. In addition, the 2D point distributions generated by CGR can be further analyzed using different methods, to extract additional structural information.

To this aim, one of the most common methods, used either standalone or in combination with other methods, is multifractal analysis, since it allows distinguishing between coding and non-coding sequences, or it can be used in phylogeny reconstruction and in investigating the clustering of protein structures. In particular, by using a CGR in which a DNA symbolic sequence is mapped onto a singular measure on the attractor of a particular iterated function system (IFS), the multifractal spectrum of the resulting measure is shown to be more sensitive for detecting dependence structures within DNA than the averaged contribution given by redundancy [16]. WTMM applied to multifractal analysis of CGR images show that the scale-invariance range of CGR edge can be extended to three orders of magnitude, and complete singularity spectra can be calculated [17]. By using CGR of protein sequences based on the detailed HP model, together with their multifractal and correlation analyses, a more precise phylogenetic tree of bacteria has been proposed [18]. CGR of randomly linked functional protein sequences used together with recurrent IFS helps to extract some biological functions of these proteins [19]. Multifractal detrended cross-correlation analysis of genome sequences using CGR shows the existence of multifractal nature and power-law correlation behavior between any pair of genome sequences [20]. A version of CGR, modified to visualize heteroplasmic mutations, used together with lacunarity analysis, reveals fractal properties of mitochondrial DNA sequences, and can quantitatively characterize Parkinson’s disease [21].

However, as we shall see below, the multifractal spectra of different point distributions can be very similar, and additional analysis is required to discriminate between such arrangements, and which can provide new insights into their structure. To address this issue, a useful approach is to use the formalism underlying analysis of small-angle scattering (SAS) [22], largely used in molecular biology for investigating the structure of complex macromolecules. Physically, when X-rays are used, SAS is based on the electrostatic interaction of the electromagnetic wave with electrons, while in the case of neutrons, it is based on their interaction with the atomic nuclei (nuclear scattering), and in ferromagnetic materials, on interaction of neutron’s magnetic moment with that of the atom (magnetic scattering). Theoretically, depending on the behavior of the SAS intensity, i.e., if we have a simple or a generalized power-law decay (i.e., a succession of maxima and minima superimposed on a simple power-law decay), one can use models specific to random (statistically self-similar) [23,24], and respectively to deterministic (exact self-similar) fractals [25].

A first step in this direction has been recently performed in Ref. [26], where SAS technique has been applied to study the structural properties of various fractal patterns generated by CGR, and an analytic expression of the corresponding SAS intensity has been derived for Sierpiński triangles. The SAS intensity from CGR has been calculated numerically by using a simplified version of the Debye formula [27]. The good agreement between numerical data and the theoretical model indicates that SAS technique can be successfully used to obtain the main structural characteristics, including the overall fractal size, scaling factor, fractal dimension, or the number of units composing the fractal, corresponding to CGRs of DNA sequences, and for which analytic expressions of intensity are not available or are hard to be derived. Such information may be useful to distinguish functional regions of DNA sequences or solving issues related to the classification of organisms. In particular, knowledge of the fractal dimension may provide information about the percentage content of coding regions, i.e., the higher the fractal dimension, the higher the coding percent [28]. In addition, the scaling factor may provide information about the relative sizes of coding regions, and which, in turn, describes the degree of self-similarity for different organisms, since it is related to the value of the fractal dimension [26].

Here, the results obtained in Ref. [26] are extended by performing a combined analysis of CGR using SAS and multifractal analysis of *Escherichia coli*, *Phospholamban* and *Mouse mitochondrial* DNA experimental data. The complementarity of the two methods is illustrated first on a model based on multiplicative deterministic cascades. A model in which both approaches can be hardly used to distinguish between various patterns is also provided, and is based on the existence of forbidden sequences in DNA. Furthermore, the two models are used as benchmarks against which the structures of the experimental data are compared in terms of the interplay between mass and surface-like fractals structures emerging in CGR. As such, it is shown that for *Escherichia coli* the structure resemble closer a random fractal, while in the case of *Phospholamban* and *Mouse mitochondrial* DNA, the corresponding structure is closer to deterministic ones.

## 2. Theoretical Background

### 2.1. Iterated Function Systems and Chaos Game Representation of DNA Sequences

The theoretical framework provided by iterated function systems (IFS) is very useful for classification and description of fractals. Mathematically, a (hyperbolic) IFS is given by a complete metric space (X,d) together with a finite set of contraction mappings $w_{n}:X\to X$, with respective contractivity factors $s_{n},n=1,2,\cdots ,N$ [29]. Using a shorthand notation, an IFS is $\left\{X;\;w_{n},n=1,2,\cdots ,N\right\}$ and $s=\max\left\{s_{n},n=1,2,\cdots ,N\right\}$. Please note that a transformation f:X→X on a metric space (X,d) is a contraction mapping if there is a constant (contractivity factor) 0≤s<1 such that $d\left(f(x),f(y)\right)\leq s\cdot d\left(x,y\right)\;\;\;\forall x,\;y\in X$.

If one considers an IFS with contractivity factor s, and $\left(H(X),h(d)\right)$ the space of nonempty compact subsets with the Hausdorff metric h(d), then the transformation W:H(X)→H(X) defined by

(1) $$W\left(B\right)=\cup_{n=1}^{N}w_{n}\left(B\right),\;\forall B\in H\left(X\right),$$

is a contraction mapping on the complete metric space $\left(H(X),h(d)\right)$ with contractivity factor *s* [29], i.e.,

(2) h(W(B),W(C))≤s·h(B,C)∀B,C∈H(X).

Its unique fixed point, A∈H(X) obeys $A=\cup_{n=1}^{N}w_{n}\left(A\right)$, is given by $A=\lim_{m\to \infty }W^{\circ m}\left(B\right)$ for any B∈H(X), and is called the *attractor* of the IFS [29].

Here, for rendering pictures of attractors we play chaos game using the random iteration algorithm. As such, one start by assigning the probability $p_{n}>0$ to $w_{n}$ for n=1,2,⋯,N, where $\sum_{n=1}^{N}p_{n}=1$. Then, a point $x_{0}\in X$ is chosen, and then recursively, next points are obtained according to:

(3) $$x_{k}\in \left\{w_{1}\left(x_{k-1}\right),w_{2}\left(x_{k-1}\right)\cdots ,w_{N}\left(x_{k-1}\right)\right\}.$$

The probability of the event $x_{k}=w_{n}\left(x_{k-1}\right)$ is $p_{n}$, and k=1,2,⋯. This generates the sequence $\left\{x_{k}:\;k=0,1,\cdots \right\}\subset X$ which converges to the attractor of IFS.

When the chaos game is played with 4 points (i.e., N=4), the matrix representation of the IFS of affine maps is:

(4) $$w_{i}\left[\begin{array}{c} x\\ y \end{array}\right]=\left[\begin{array}{cc} a_{i} & b_{i}\\ c_{i} & d_{i} \end{array}\right]\left[\begin{array}{c} x\\ y \end{array}\right]+\left[\begin{array}{c} e_{i}\\ f_{i} \end{array}\right]$$

where the coefficients $a_{i},b_{i},c_{i},d_{i},e_{i},f_{i}$ with i=1,2,3,4. For a uniformly filled square, they are given in Table 1.

To display visually the underlying structure of the attractor resulting from a string of four letters, Jeffrey proposed [14] to construct a square with vertices “A”, “C”, “G” and “T”, and to control the chaos game, not with the probabilities $p_{i}$ (see Table 1), but with the DNA sequence. In particular, to choose the next point, one could use the next base in the DNA sequence. For a sequence of three letters “CGT”, the algorithm translates as follows: first, plot “C” at half distance between the center of the ACGT square and “C” vertex, then, the next base “G” is plotted at half distance between the previous point and “G” vertex, and finally, the last bases “T” is plotted at half distance between the previous point and the “T” vertex.

### 2.2. Fractals and Multifractals

Mathematically, description of fractals is based on concepts from measures theory [30], and a rigorous definition has been introduced by Hausdorff in Ref. [31]. This involves a subset S of the *n*-dimensional Euclidean space, and let us consider that $\left\{C_{i}\right\}$ is a cover of S with $c_{i}=\mathrm{diam}\left(C_{i}\right)\leq s$ and s∈ S. Then, the Hausdorff measure $m^{\alpha }\left(S\right)$ is given by taking the infimum over all possible coverings, i.e.,

(5) $$m^{\alpha }\left(S\right)=\lim_{s\to 0}\inf_{\left\{C_{i}\right\}}\sum_{i}c_{i}^{\alpha },\;\;\;\mathrm{with}\;\;\;\alpha \in R^{+},$$

and thus, the fractal dimension *D* can be written as:

(6) $$D\equiv \inf\left\{\alpha :m^{\alpha }\left(S\right)=0\right\}=\sup\left\{\alpha :m^{\alpha }\left(S\right)=+\infty \right\}.$$

This corresponds to the value of α for which the Hausdorff measure changes from zero to infinity. When α=D, $m^{\alpha }\left(S\right)$ can have arbitrary values within this range.

However, for most practical purposes, it is very difficult to use Equation (6) for determination of the fractal dimension. To avoid this issue, another approach is to determine the variation of the fractal measure M, inside a sphere of dimension *n* and radius *r* centered on the fractal. For fractal systems, one can write a mass–radius relation of the type [32]:

(7) $$M\left(r\right)=A\left(r\right)r^{D},$$

where $\lim_{r\to \infty }\log A\left(r\right)/\log r\to 0$. The above equation plays a central role in development of the concepts used thereafter, since it allows us to describe mass and surface fractals, and provide analytical expression for fractal dimensions for a large class of fractals.

Thus, let us consider further a fractal of size *L* composed of balls of size *a*. Then, the number of balls enclosed by the imaginary sphere of radius *r* with a ball in the center, is given by [32]:

(8) $$N\left(r\right)\propto {\left(r/a\right)}^{D}\propto r^{D},$$

with l≲r≲L. If the fractal is a line, one has D=1, for a smooth surface, D=2, while for a regular Euclidean 3D object, D=3.

For fractals with a scaling factor $\beta_{s}$, one can use the property that at first iteration the fractal consists of *k* copies of itself, each of size $\beta_{s}L$, and write that [32]:

(9) $$M\left(L\right)=kM\left(\beta_{s}L\right).$$

Then, by using Equation (7), one obtains:

(10) $$k\beta_{s}^{D_{m}}=1,$$

which can be used to obtain the fractal dimension *D*. For fractals with multiple scaling factors $\beta_{si}$ and $k_{i}$ copies with i=1,⋯,n, Equation (9) is rewritten as:

(11) $$\sum_{i=1}^{n}k_{i}\beta_{si}^{D_{m}}=1.$$

Similarly, for surface fractals one can write:

(12) $$S\left(r\right)=S_{0}r^{2-D_{s}},$$

where S(r) represents the area between the boundary of the (rough) surface and the envelope of all spheres of radius *r* centered on the boundary. Here, $S_{0}$ is a constant, which is the surface area itself for a smooth surface, i.e., when $D_{s}=2$.

The fractal dimensions *D* which appear in the above relations, are equivalent to the box-counting dimension and describe fractals with a single scaling factor. However, when several scaling factors are present, a more detailed description is required, and can be achieved by using the multifractal formalism [33,34], where one considers an object *S* covered by a grid of boxes $B_{i}\left(l\right)$ of size *l*. By considering that the measure determined by the probability of hitting the object in the box $B_{i}$ is μ(B), the number of covered boxes *N* at resolution *l* is $N\propto 1/l^{2}$, and thus one can write [35]:

(13) $$Z_{s}\left(l\right)=\sum_{i=1}^{N}p_{i}^{s}\left(l\right),$$

where *Z* is called the “partition function” and has a power-law behavior when l→0 and N→∞, so that $Z_{s}\propto l^{D_{s}\left(s-1\right)}$. Here, *i* denotes each individual box, and $p_{i}=\mu \left(B\right)$ with $\sum_{i=1}^{N}p_{i}=1$, are the hitting probabilities. Then, the generalized dimension spectrum is given by [35]:

(14) $$D_{s}\equiv \frac{1}{s-1}\lim_{l\to 0}\frac{\ln Z_{s}\left(l\right)}{\ln l}.$$

By considering the ratio $p_{i}\equiv N_{i}\left(l\right)/N$, which gives the relative weight of the *i*-th box, one can write:

(15) $$D_{s}=\frac{1}{s-1}\lim_{l\to 0}\frac{\ln\sum_{i=1}^{N}p_{i}^{s}\left(l\right)}{\ln l}.$$

The generalized dimension spectrum is a monotonically decreasing function, with horizontal asymptotes at $\alpha_{max}=\lim_{q\to -\infty }D_{s}$ and $\alpha_{min}=\lim_{q\to \infty }D_{s}$. Their values can be used to describe the heterogeneity, i.e., if $\alpha_{max}\neq \alpha_{min}$ the fractal is heterogeneous (multifractal), and homogeneous otherwise.

An equivalent representation of generalized dimension spectrum is provided by f(α) spectrum, which gives a mathematically precise and intuitive description of the multifractal measure in terms of interwoven sets, with singularity strength α, and Hausdorff dimension f(α). Without going into the details of derivation here, it can be shown that the generalized dimension and f(α) spectra are related through a Legendre transform [36], i.e.,

(16) f(α)=sα(s)−τ(s),

where α(s)=dτ(s)/ds and $\tau \left(s\right)=\left(s-1\right)D_{s}$.

In the remaining of the paper, Equations (15) and (16) are used to characterize the multifractal properties of point distributions generated by CGR described in the previous section. To illustrate the method, analytically solvable models given by multiplicative deterministic cascades are first analyzed in Section 3.1.1.

### 2.3. Small-Angle Scattering

One considers here a two-phase approximation in which microscopic scattering objects with scattering length $b_{j}$ have the scattering length density (SLD) $\rho_{s}\left(r\right)=\sum_{j}b_{j}\delta \left(r-c_{j}\right)$, where $c_{j}$ are the position vectors of the objects. The differential elastic cross section is defined by $d\sigma /d\Omega ={\left|A_{t}\left(q\right)\right|}^{2}$, where q is the scattering vector. For a three-dimensional object $A_{t}\left(q\right)=\int_{V^{{}^{\prime }}}\rho_{s}\left(r\right)\exp\left(iq\cdot r\right)dr$ is the total scattering amplitude and $V^{{}^{\prime }}$ is the irradiated volume. When the objects are embedded in a solid matrix of SLD $\rho_{0}$, then the scattering contrast is defined by $\Delta \rho =\rho -\rho_{0}$, and the total scattering intensity can be written as [22]:

(17) $$I\left(q\right)\equiv \frac{1}{V^{{}^{\prime }}}\frac{d\sigma }{d\Omega }=c{\left|\Delta \rho \right|}^{2}V^{2}\langle {\left|F(q)\right|}^{2}\rangle ,$$

where *c* is the concentration of objects, *V* is their volume, and $F\left(q\right)\equiv \left(1/V\right)\int_{V}\exp\left(-iq\cdot r\right)dr$ is the normalized form factor, with F(0)=1, and the symbol $\langle \cdots \rangle$ denotes the ensemble averaging over all orientations. In what follows, since the object are two-dimensional ACGT squares, the volume *V* in Equation (17) shall be replaced by the corresponding surface area. Please note that the above averaging procedure allows the rotation of the squares in three-dimensional space, with equal probability.

Since the positions of the points are generated by CGR, we can start with the Debye formula to calculate the scattering intensity, and thus Equation (17) can be written as [27]:

(18) $$I\left(q\right)=NI_{s}\left(q\right)+2F_{s}{\left(q\right)}^{2}\sum_{i=1}^{N-1}\sum_{j=i+1}^{N}\frac{\sin qr_{ij}}{qr_{ij}},$$

where $I_{s}\left(q\right)=1$ is the intensity scattered by each point, *q* is the magnitude of the scattering vector q, and $r_{ij}$ is the distance between arbitrary points *i* and *j*. When the number of points exceeds a few thousand, the computation of the term $\sin\left(qr_{ij}\right)/\left(qr_{ij}\right)$ is very time consuming, and thus it is handled via a pair-distance histogram g(r), with a bin-width commensurate with the experimental resolution [37]. Therefore Equation (18) becomes:

(19) $$I^{D}\left(q\right)=N+2\sum_{i=1}^{M_{\mathrm{bins}}}g\left(r_{i}\right)\frac{\sin qr_{i}}{qr_{i}},$$

where $M_{\mathrm{bins}}$ is the number of bins, and $g(r_{i})$ is the pair-distance histogram at pair-distance $r_{i}$. The latter quantity is calculated from the positions of points inside the ACGT square.

## 3. Results and Discussion

### 3.1. Analysis of Theoretical Models

To illustrate the complementarity between SAS and multifractal techniques in analyzing point distributions, one considers a model of multiplicative deterministic cascades. This is followed by a second model, based on forbidden sequences in DNA, and which shows that both the SAS and multifractal spectra can hardly be used to differentiate between various structures.

#### 3.1.1. Multiplicative Deterministic Cascades

**Figure 1:**
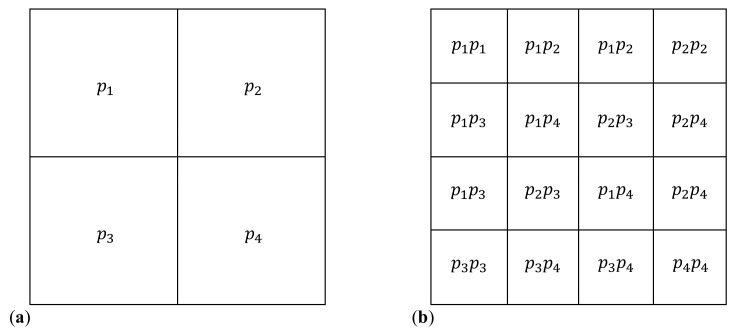
The initiator (**a**) and generator (**b**) of the multifractal lattice. The quantities $p_{1},p_{2},p_{3}$ and $p_{4}$ represent the probabilities associated with their corresponding cells in the lattice.

The multiplicative deterministic cascade model is constructed by dividing a square into for equal squares. To each of the subsquare, one assigns the probabilities $p_{i}\in \left[0,1\right]$, with i=1,2,3,4. This is called the first iteration, and it is denoted n=1 (Figure 1a). Then, at second iteration (n=2), each of the four subsquares is further divided in four squares, and to each of them are assigned probabilities given by the Kronecker product of the matrices with the elements $p_{i}$ placed as in Figure 1a. The results are shown in Figure 1b. At third iteration, one perform a similar division of squares, and to each of them one assigns the probabilities given by the Kronecker product of the matrices with as elements the probabilities from iteration n=1, and respectively from n=2. The multiplicative cascade model is obtained in the limit of high number of iteration, and the distribution of square values therefore depends on the initial choices of probabilities $p_{i}$. This model is similar to the model for the displacement of a viscous fluid by a nonviscous fluid in a porous medium, developed in Ref. [38]. However, in this reference, at n=2 the probabilities $p_{i}$ associated with each such division are multiplied in random order by probabilities $p_{i}$. The same random assignment of probabilities is kept for subsequent iterations, thus leading to a multiplicative random cascade model.

**Figure 2:**
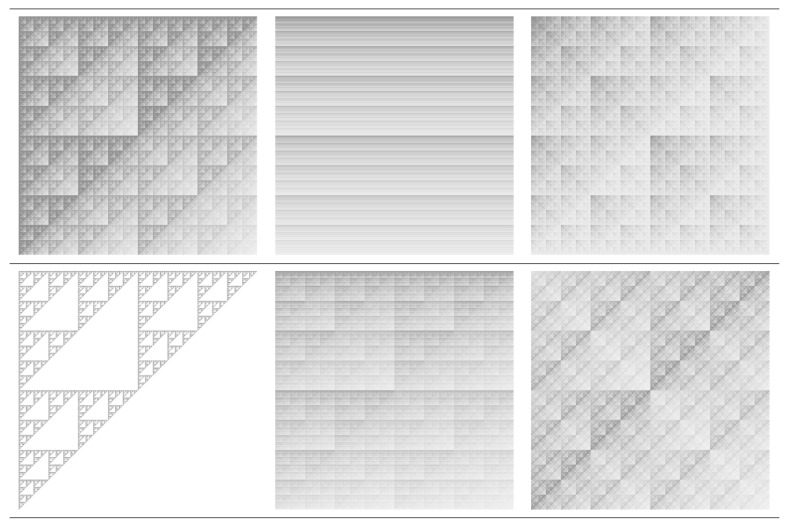
Six configurations M1, M2, M3, M4, M5, and M6 of the multiplicative deterministic cascade model, at iteration number n=11. Upper part: M1, M2, M3 (from left to right). Lower part: M4, M5, M6 (from left to right). See Table 1 for the corresponding probabilities $p_{1},p_{2},p_{3}$ and $p_{4}$. The model M7 is not shown here, since it corresponds to a uniformly filled square.

Figure 2 shows several realizations of the multiplicative deterministic cascades model, in which the probabilities $p_{i}$ are given in Table 2. The common feature is the presence of a deterministic pattern, in which exact copies of the fractal appear at various scales. The structure of the pattern is very rich, and includes stripes of lines (model M2), single scale Sierpiński gaskets (model M4), or a clear superposition of Sierpiński gaskets (model M4).

For these models of multiplicative cascades, it can be shown that the generalized dimension spectrum has an analytic expression given by [39]:

(20) $$D_{s}=\frac{1}{1-s}\log_{2}\left(f_{1}^{s}+f_{2}^{s}+f_{3}^{s}+f_{4}^{s}\right),$$

where $f_{j}=p_{j}/\sum_{i=1}^{4}p_{i}$, and j=1,2,3,4.

**Figure 3:**
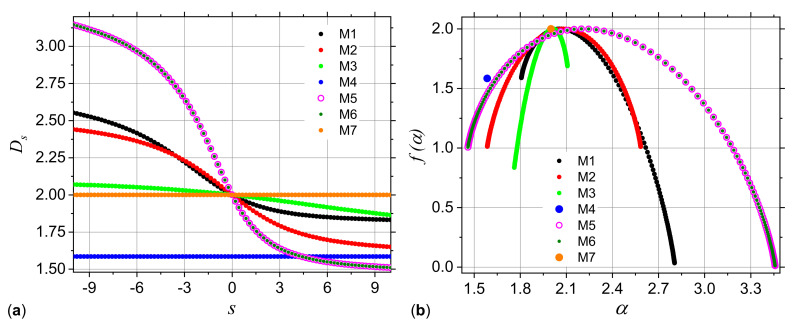
Generalized dimensions $D_{s}$ (**a**) and f(α) spectra (**b**) for the configurations M1, M2, M3, M4, M5, M6 and M7. See Table 1 for the corresponding probabilities $p_{1},p_{2},p_{3}$ and $p_{4}$.

The corresponding dimension spectra are presented in Figure 3a. The results clearly show that the spectra of models M1, M2, M3, M5 and M6 have a decreasing behavior, indicating that the corresponding structures are multifractals. However, for model M4 the spectrum is constant and indicates a simple fractal structure (Sierpiński gasket) with fractal dimension about 1.58, as expected. Also, in the case of model M7 (uniform square) the fractal dimension 2 is recovered. Please note that for models M5 and M6 the dimension spectra coincide for all *s*. They are also the most heterogeneous structures, since the difference $\alpha_{\min}-\alpha_{\max}≃3.45-1.48=1.97$ is maximum, as compared to the other models.

The f(α) spectra are calculated by using Equation (16), and the results are presented in Figure 3b. Generally, these spectra are convex functions with a single maximum at $\alpha =\alpha_{0}$. This gives the most frequent value of the scaling indices α, and appears at s=0, where $f\left(\alpha \right)=D_{0}$ [39]. Also, the scaling indices take vales in the finite range $\left[\alpha_{\min},\alpha_{\max}\right]$. The smaller the length of this interval, the more homogeneous the structure. In particular, for models M4 and M7, the spectra degenerate into a single point at f(α)≃1.58 and respectively f(α)=2. Please note that for models M1, M3, M5 and M6, the f(α) spectra are asymmetric with respect to their maxima. Lower values of f(α) for s<0 (models M1, M5 and M6) indicate a higher influence of the lowest values of the fractal measure in the spectral complexity. Similarly, the appearance of lower values of f(α) for q>0 (model M3) indicate a higher influence of the lowest values of the fractal measure [40].

**Figure 4:**
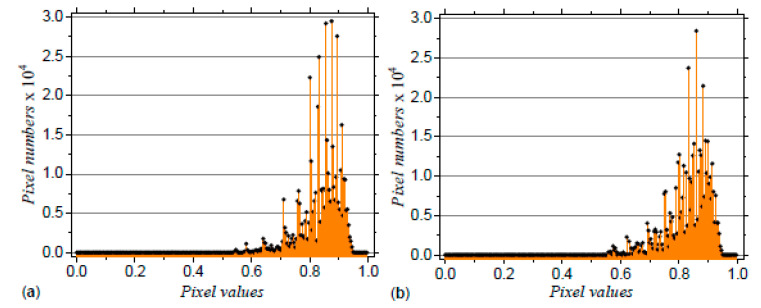
Histograms of the pixel levels in the images corresponding to models M5 (**a**) and M6 (**b**), shown in Figure 2 lower part.

In the following one obtains the SAS from models M5 and M6. To this aim, the coordinates of the points needs to be extracted from Figure 2 and used as input in Debye equation (Equation (19)). However, a general property of such images is that the distribution of grey levels is concentrated within a short range, and thus image binarization at various thresholds becomes impracticable. Figure 4 shows this situation for models M5 and M6, where the number of pixels are mostly concentrated in the 0.7 ÷ 0.95 range. Despite this local pixel concentration, their distribution is different, and this indicate that SAS technique can distinguish between the two structures.

**Figure 5:**
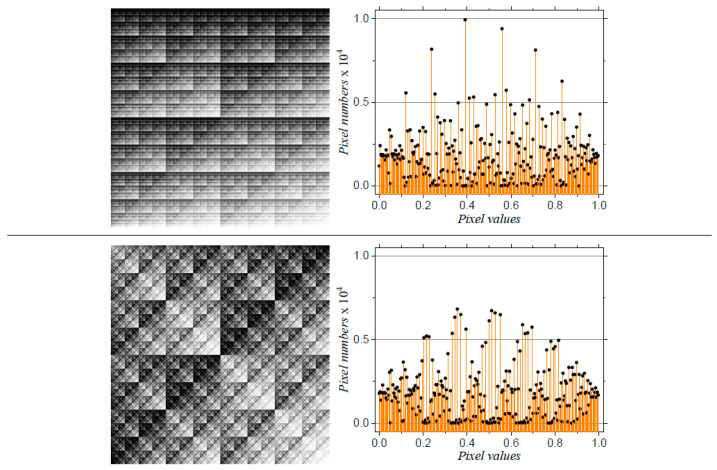
Images with equalized histograms for configurations M5 (upper-left corner) and M6 (low-left corner). Upper- and lower-right corers: the corresponding histograms.

**Figure 6:**
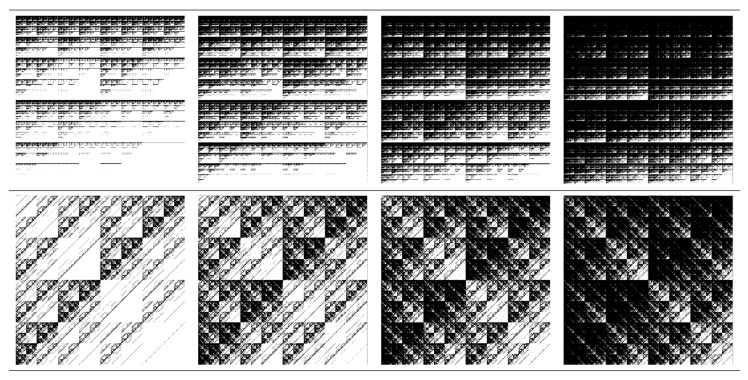
Binarized images of configurations M5 (upper-row) and M6 (lower-row) at different thresholds *t*. Columns from left to right: t=0.2, t=0.4, t=0.6, t=0.8.

Therefore, an equalization of histograms such that they span the entire range from 0 (black) to 1 (white) needs first to be performed. This is done in Figure 5, which shows both the transformed image and the corresponding pixel distribution (upper part—model M5, and lower part—model M6). The histogram distribution of the models show a periodicity specific to multiplicative deterministic cascade structures, since the grey levels alternate in a regular fashion. The heights of the single maxima (for model M5) and of the groups of maxima (for model M6) follow a parabola-like behavior over the entire range of pixel values. These features now allows the analysis of the transformed images at various thresholds *t*. Figure 6 shows the corresponding binarized images for models M5 (upper part) and M6 (lower part) at thresholds 0.2, 0.4, 0.6 and 0.8 (from left to right).

**Figure 7:**
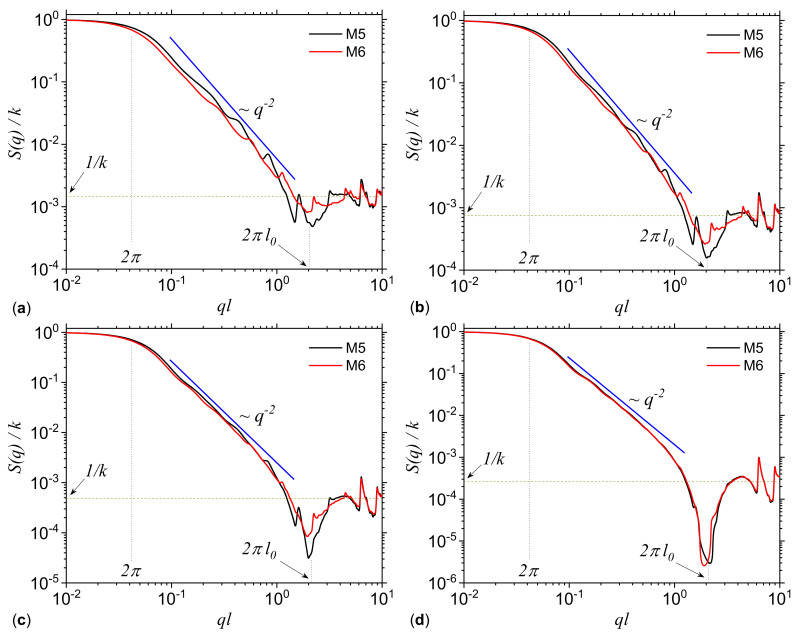
Structure factor (Equation (19)) corresponding to configurations M5 and M6, at various thresholds *t*. (**a**) t=0.2. (**b**) t=0.4. (**c**) t=0.6. (**d**) t=0.8. Here, *l* is the overall size of the fractal in pixels, *k* is the number of basic units composing the fractal, and $l_{0}$ is the pixel size.

The structure factor of the point distributions in Figure 6 is calculated by using Equation (19) and the results are shown in Figure 7. The main feature of the scattering curves in all cases is the presence of three structural regimes: $S\left(q\right)\propto q^{0}$ at ql≲2π (Guinier region), $S\left(q\right)\propto q^{-D}$, with D≃2 at $2\pi ≲ql≲2\pi l_{0}$ (fractal region), and S(q)≃1/k at $2\pi l_{0}≲ql$ (asymptotic region). Here, *l* is the overall fractal size (in pixels), *k* is the number of points composing the fractal, and $l_{0}$ is the pixel size. The length of the Guinier region provides information about the overall fractal size and they are very similar at each threshold, since the dimensions of squares are the same. In the fractal region one can see a generalized power-law decay (GPLD), i.e., the presence of a succession of maxima and minima superimposed on a simple power-law decay. A GPLD is characteristic to deterministic fractals [25] and can be used, as we shall see below, in extracting the fractal iteration number and the scaling factor.

The differences between scattering curves for models M5 and M6 are clearly visible at smaller values of the thresholds, and less visible at higher values, in particular, at t=0.8 the differences are practically indistinguishable. This is because by increasing *t* the distribution of points becomes more uniform (see last column in Figure 6), and the deterministic character cease to dominate. Consequently, the maxima and minima are smoothed to the extent that the resulting curves coincide. Also, the scattering exponent D≃2 is kept in all cases ant it coincides with the numerical values of $D_{0}$ given by multifractal spectra (see Figure 3). Finally, in the asymptotic region one can see that the total number of pixels *k* increases with *t* (since the ratio 1/k decreases), and this can be used to obtain the total number of points present in the fractal.

#### 3.1.2. Missing Sequences Models

**Figure 8:**
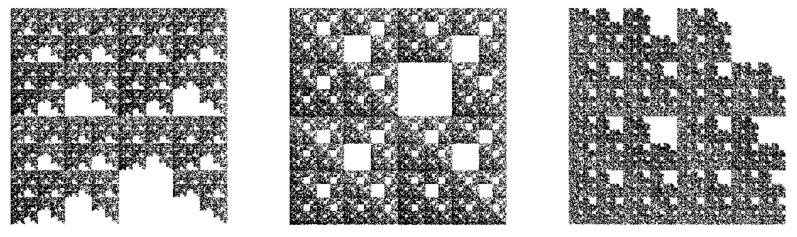
CGR in the “ACGT” square, with $A=\left(0,0\right)$, $T=\left(1/2,0\right)$, $G=\left(1/2,1/2\right)$, $C=\left(0,1/2\right)$ played with $8\times 10^{4}$ points. Left part: “AT” moves are forbidden (model TR12). Middle part: “AG” moves are forbidden (model TR13). Right part: “GG” moves are forbidden (model TR33).

Once a complete genome is available, a natural question which arise is whether there are subsequences of short strings of letters “A”, “C”, “G” or “T” that are missing, since this may reveal some biological meanings such as evolutionary relatedness of species [41]. To this aim, important steps have been performed in Ref. [42], where sequences are transformed into portraits, and thus the missing sequences can be visualized. Although this method has also the advantage of providing analytic expressions of the fractal dimensions of the patterns emerging from this visualization scheme [43], here CGR shall be used since in the case of missing of certain types of string composed on 2-contiguous letters, the resulting pattern has an obvious fractal structure (see Figure 8), while the fractal dimension can be extracted from either SAS or multifractal spectra, as discussed in the previous section. Moreover, by establishing relations between DNA sequences with missing subsequences, and the generalized Cantor sets, such as those presented in Ref. [44], the possibility of using SAS technique in analyzing structures with missing subsequences can be greatly extended, since theoretical expressions for SAS intensities are already available for some classes of generalized Cantor fractals [45].

In the following, one considers a random sequence of length 8 × 10${}^{4}$ consisting from the four letters “A”, “C”, “G” or “T”, but that has never “A” followed by “T” (model TR12), “A” followed by “G” (model TR13), or “G” followed by “G” (model TR33). The corresponding structures obtained from CGR are shown in Figure 8. The resulting patterns has fractal properties in which the complementary structure, i.e., white regions, have various geometrical shapes (model dependent) and with sizes following a power-law distribution. The simplest complementary structure is shown in Figure 8 (middle part), and which consists from one square of edge length 1/8, $4^{1}$ squares of edge lengths 1/16, $4^{2}$ squares of edge lengths 1/32 and so on. Such a distribution is specific to surface-like fractals and has important implications in behavior of the corresponding scattering curves. In particular, when the associated measure is multifractal, then the SAS intensity shows a not only a single mass fractal region, as in Figure 7, but a *succession* of mass fractal regime followed by a surface fractal one [46].

**Figure 9:**
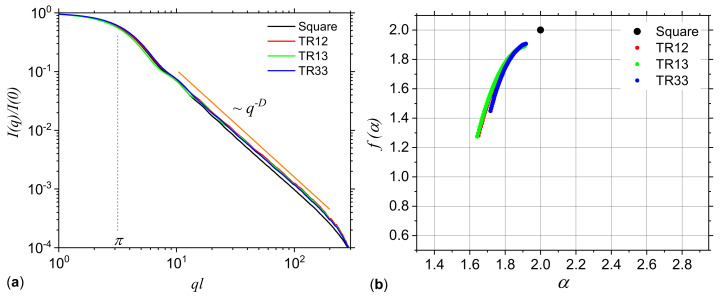
Scattering intensities (**a**) and f(α) spectra (**b**) for models TR12, TR13 and TR33. *l* is the overall size of the “ACGT” square. The data for a uniform square are included for comparison.

Since for all three models, both the number of complementary regions forming the surface fractal, as well as their scaling factor are unchanged, one may expect that the corresponding SAS curves from these models to be quite similar. This is confirmed numerically in Figure 9a, which shows that the SAS curves decay $\propto q^{-D}$, with D≲2 in the fractal regime, and they are hardly distinguishable. For comparison, the SAS intensity of a square, i.e., D=2 is also included (black curve). The absence of maxima and minima in the fractal regime reflect a nearly uniform arrangement of the points, resulting from the random process used in generating their positions. The f(α)-spectra for these models are shown in Figure 9b. They are confined in a narrow range of α values, i.e., 1.62≲α≲1.95 for models TR12 and TR13, and 1.72≲α≲1.95 for model TR33, thus indicating that the multifractal nature is not so pronounced as in the case of multiplicative cascade models (see also Figure 3b). Also, for comparison, Figure 9b includes the f(α)-spectrum of a uniform distribution of points in the “ACGT” square, which is an Euclidean object. This is represented as a single point (black) of coordinates $\left(2,2\right)$.

The results in Figure 9 illustrate that generally, SAS and $f\left(\alpha \right)$ can hardly be used to perform a clear differentiation between such structures. This issue may be eventually addressed by establishing first the relationship with the corresponding generalized Cantor set, as performed in Ref. [44], followed then by calculating the corresponding SAS intensities.

### 3.2. Application to DNA Sequences: *Phospholamban*, *Mouse mitochondrion* and *Escherichia coli*

**Figure 10:**
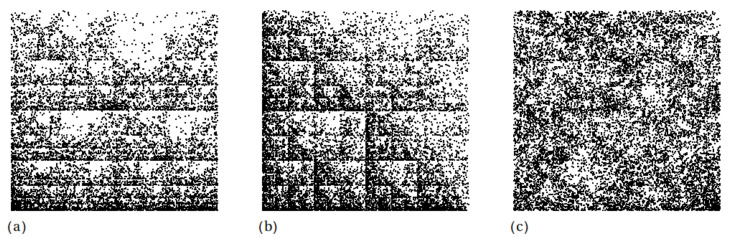
CGR in the “ACGT” square, with $A=\left(0,0\right)$, $T=\left(1/2,0\right)$, $G=\left(1/2,1/2\right)$, $C=\left(0,1/2\right)$ for *Phospholamban*, GenBank ID: 5350 (**a**), *Mouse mitochondrion*, GenBank ID: 342520 (**b**), and *Escherichia coli* (**c**), NCBI Accession Version NZ_BJSS01000161.

The CGR followed by SAS and multifractal analysis is illustrated on *Phospholamban* [homo sapiens (human); GenBank ID: 5350] with 12,116 bp (Figure 10a), *Mouse mitochondria*, complete genome (GenBank ID: 342520) with 16,295 bp (Figure 10b), and *Escherichia coli* O145:H28 strain 162,405 sequence161, whole genome shotgun sequence (NCBI Accession Version NZ_BJSS01000161) with 15,000 bp (Figure 10c).

The protein encoded by *Phospholamban* is found as a pentamer, is a major substrate for the cAMP-dependent protein kinase in cardiac muscle, and is an inhibitor of cardiac muscle sarcoplasmic reticulum Ca(2${}^{+}$)-ATPase in the unphosphorylated state [47]. However, inhibition is relieved upon phosphorylation of the protein, and subsequent activation of the Ca(2${}^{+}$) pump leads to enhanced muscle relaxation rates, thereby contributing to the inotropic response elicited in heart by beta-agonists. The encoded protein is a key regulator of cardiac diastolic function. Mutations in this gene are a cause of inherited human dilated cardiomyopathy with refractory congestive heart failure, and familial hypertrophic cardiomyopathy [47].

In the case of *Mouse mitochondrion*, the genome displays exceptional economy of organization, with tRNA genes interspersed between rRNA and protein-coding genes with zero or few non-coding nucleotides between coding sequences [48]. The genome is highly homologous in overall sequence as well as the organization to human mitochondrial DNA, and an important feature in that the translational start codon is AUN, with any of the four nucleotides in the third position, whereas the only translational stop codon is the orthodox UAA [48].

O145:H28 is one of the major non-O157 Shiga toxin (Stx)-producing *Escherichia coli* (STEC) lineages that causes severe diseases and is frequently isolated from humans, animals and foods [49]. Highly dynamic features of prophages and plasmids in the diversification of the STEC lineage, and the differential dynamics and impacts of these mobile genetic elements on the pangenome and virulence factor repertoire among O145:H28 strains [50].

The CGR of *Phospholmban* and *Mouse mitochondrion* in Figure 10 resemble more closer a deterministic-like fractal structure, in which a pattern repeats itself at increasingly smaller scales. In particular, the structure of *Phospholamban* resemble, in a first approximation, the structure of model TR12 shown in Figure 8.

Note that in “CG” sub-quadrant of CGR from *Phospholamban*, i.e., in the upper-left region of the G-quadrant in Figure 10, the number of points are relatively much smaller as compared to other sub-quadrants. This indicates a paucity of subsequences ending in “CG” [14]. Similarly, the high number of points in “TT”, “AT”, “TA” and “AA” sub-quadrants, indicates a higher number of subsequences ending with these suffixes.

However, for *Mouse mitochondrion*, the white regions specific to model TR12, are partially populated with points, while their overall density is higher at the bottom. The paucity of subsequences ending in “CG” is still preserved, but however, the “AA” sub-sequence seems to be predominant. As it shall be seen below, this has important consequences on the behavior of SAS and f(α)-spectra. A somehow similar arrangement can be seen also for *Mouse mitochondrion*, where a higher density of points is formed within the “ATC” triangle. The structure of *Escherichia coli* is characterized by the coexistence of regions with different densities, distributed more uniformly, as compared to *Phospholamban* and *Mouse mitochondrion*.

**Figure 11:**
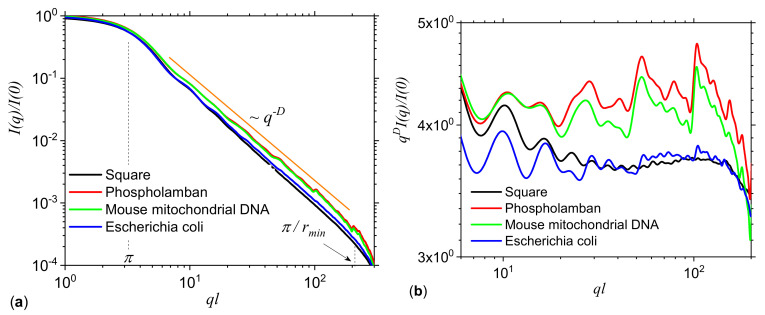
Scattering intensities I(q) (**a**) and $q^{D}I\left(q\right)$ (**b**) of *Phospholamban* (red), *Mouse mitochondrion* DNA (green), and *Escherichia coli* (blue). For comparison, the scattering intensity of a uniform distribution of points within a square is shown. *l* is the overall size of the “ACGT” square, $r_{min}$ is the minimum scale on which the samples have a fractal behavior, and *D* is the fractal dimension (see text for details).

Figure 11a shows the SAS intensities from *Phospholamban*, *Mouse mitochondrion* and *Escherichia coli* calculated with Equation (19). For comparison, the scattering curve of a uniformly filled square of the same size as the “ACGT” square is included. The results show the presence of the fractal region at $\pi ≲ql≲\pi /r_{\min}$, where *l* is the overall size of the square (360 × 360 pixels${}^{2}$), and $r_{\min}$ is related to the smallest size of the pixels in the image. The common feature is the power-law decay with scattering exponent D≲2, revealing a mass fractal structure in all three cases. However, for *Phospholamban* and *Mouse mitochondrion*, the scattering curve has a generalized power-law decay (see Section 1). To investigate this behavior in more details, Figure 11b shows the curves $q^{D}I\left(q\right)/I\left(0\right)$, which reveal more clearly the periodicity of *Phospholamban* and *Mouse mitochondrion* in the fractal regime. This is a signature of their deterministic nature, as indicated also by their structures shown in Figure 10a,b. Then, the periodicity and the number of minima can be related to the scaling factor β and to the number of iterations [25]. In particular, the curve $q^{D}I\left(q\right)/I\left(0\right)$ is approximately log-periodic with the period equal to the inverse scaling factor, and thus β≃1/2, while the number of iterations is equal to three. Please note that the value of the scaling factor is related to the CGR algorithm, and thus if one plot a given point, not at half distance between the previous point and the corresponding vertex, but at another fraction, or even using a 3D CGR as described in Ref. [51], one expects to recover also the corresponding scaling factor. Therefore, a SAS analysis could be employed to check the nature of the algorithm used to generate the points distribution.

Symmetry properties occurring in the “ACGT” square can also be revealed by performing a structural analysis in real space. Thus, by taking the Fourier transform of Equation (19), one obtains the corresponding pair-distance distribution function (pddf) p(r), which gives the probability density of finding the distance *r* between two arbitrary points. For fractal structures, pddf can be used to reveal the fractal scaling factors, since it is characterized by a succession of groups of maxima and minima distributed periodically on a double logarithmic scale [45,52].

**Figure 12:**
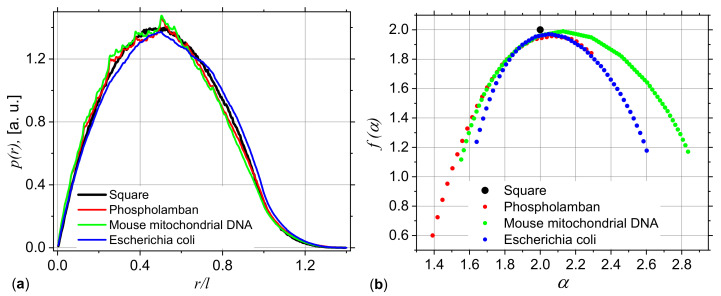
Pddf (**a**) and f(α) spectra (**b**) for *Phospholamban Mouse mitochondrion* DNA and *Escherichia coli*. The data for a uniform square are included for comparison.

Figure 12a shows the pddf of *Phospholamban*, *Mouse mitochondrion* and *Escherichia coli*. For comparison, the pddf of a uniform distribution of points is included. The common feature is that all curves follow the same trend as that given by the uniform distribution, thus reflecting the symmetry properties of the square. In addition, the most common distances occur at r/l≃0.5, i.e., at half the edge length of “ACGT” square, while the maximum distance between two arbitrary points occurs at r/l≃1.4. However, the pddf of *Phospholamban* and *Mouse mitochondrion* are characterized by small maxima and minima in the form of wriggles, in the range 0.15≲r/r≲0.8. This is the result of their deterministic nature and correspond to the generalized power-law decays occurring in Figure 11a,b in the fractal region. Finally, the pddf of *Escherichia coli* shows slight variations in the range 0.2≲r/l≲1. In particular, for 0.2≲r/l≲0.7 the pddf has smaller values as compared with the pddf of the uniform distribution of points, thus indicating the presence of more rarefied regions. However, for 0.7≲r/l≲1, the pddf is slightly higher as compared to the pddf of the uniform distribution, thus indicating the presence of more dense regions (see also Figure 10c).

The non-uniform points density distribution in the CGR for these genomes is also reflected in Figure 12b, which shows that all structures are characterized by a multifractal structure. In particular, the *Phospholamban* appears to have the most heterogeneous distributions, reflected by the highest range of α values spanned by f(α) curve. Please note that the maxima of all spectra occurs at f(α)≲2, in agreement with the values of fractal dimensions obtained from the scattering exponents in the fractal regime, shown in Figure 11a.

## 4. Conclusions

In this work SAS and multifractal techniques are employed to perform a structural analysis of CGR of DNA sequences. To address the complementarity of the two techniques, they are applied first to two theoretical models, i.e., to a deterministic multiplicative cascades and to missing sequences, and then to genome data of *Phospholamban*, *Mouse mitochondrion* and *Escherichia coli*.

The deterministic multiplicative cascades model may serve as a benchmark against which other structures can be compared, since it provides an analytical expression for the generalized dimension spectra. It has been shown that within this model, multifractal analysis may not always differentiate between the generalized dimension, and consequently between f(α) spectra (Figure 3), but however, when an appropriate threshold is used, SAS may distinguish between such structures. The differentiation is based on the behavior of the scattering curves in the fractal regimes and on the specific way, i.e., amplitude, periodicity and number of most pronounced maxima and minima, change with the magnitude of the scattering vector (Figure 7).

The missing sequences model addresses the question of whether there are subsequences of short strings of letters (“A”, “C”, “G” and “T”) that are missing. Here, a detailed analysis is performed on structures in which “A” is never followed by “T” or “G”, and when “G” is never followed by “G”. It is shown that SAS and multifractal techniques can hardly differentiate between such structures, since their corresponding spectra coincide (Figure 9). This may be attributed to the interplay between mass and surface-like fractals appearing in the “ACGT” square. While the mass fractals consist from the points resulted from CGR, surface-like fractals are formed by the power-law distribution of unoccupied regions (Figure 8).

Analysis of DNA sequences of *Phospholamban*, *Mouse mitochondrion* and *Escherichia coli* reveals that both SAS and multifractal techniques can be used to distinguish between structures (Figure 11 and Figure 12), as well as to extract additional structural information. SAS shows that *Phospholamban* and *Mouse mitochondrion* DNA generate a more ordered arrangement in the CGR, as compared to *Escherichia coli*. Figure 10 shows the corresponding arrangements. In the former case, these resemble closer deterministic structures, and in addition to the fractal dimension, it allows us to obtain the scaling factor (from the log-periodicity of minima in the curve $q^{D}I\left(q\right)/I\left(0\right)$) and the fractal iteration number (from the number of these minima; Figure 9b). *Escherichia coli* generates a more uniform distribution but still with some density fluctuations (Figure 10c). For such a structure, SAS is characterized by the absence of clear periodicity of maxima and minima in the fractal regions (Figure 11b). The heterogeneity of such structures is clearly revealed also in the f(α) spectra (Figure 12b), and which show that *Phospholamban* and *Mouse mitochondrion* have the highest structural heterogeneity.

The analysis performed in this work provides good insights on the discriminating power of both SAS and multifractal formalism, and gives useful structural information which may help to distinguish between coding and non-coding sequences in DNA, phylogeny reconstruction or clustering of protein structures. In particular, the fractal dimension *D* of coding segments has smaller values as compared to non-coding segments [53]. This can be used to quantify the excess of short runs and the deficit of long runs of weak and of strong hydrogen bases in coding sequences. The dimensions $D_{-2},D_{-1}$ and $D_{1}$ from the multifractal spectra (Equation (15)) can be used to perform a classification of bacteria by assigning to each sequence a point in 2D and 3D spaces $\left(D_{-1},D_{1}\right)$, and respectively $\left(D_{1},D_{1},D_{-2}\right)$. In this representation, bacteria that are close phylogenetically, are almost close in these spaces [54].

## Figures and Tables

**Table 1:** The coefficients $a_{i},b_{i},c_{i},d_{i},e_{i},f_{i}$ of the affine transforms $w_{n}$ (first column) in Equation (4), and the probabilities $p_{i}$ (last column) associated with each affine map.

*w*	a	b	c	d	e	f	p
1	1/2	0	0	1/2	0	0	1/4
2	1/2	0	0	1/2	0	1/2	1/4
3	1/2	0	0	1/2	1/2	0	1/4
4	1/2	0	0	1/2	1/2	1/2	1/4

**Table 2:** The probabilities $p_{i}$ used to generate the models M1, M2, M3, M4, M5 and M6 in Figure 2.

Model	$p_{1}$	$p_{2}$	$p_{3}$	$p_{4}$
M1	1	1	1	0.5
M2	1	1	0.5	0.5
M3	1	0.75	0.75	0.75
M4	1	1	1	0
M5	1	1	0.5	0.25
M6	0.5	1	1	0.25
M7	1	1	1	1
